# Climate change adaptation benefits of potential conservation partnerships

**DOI:** 10.1371/journal.pone.0191468

**Published:** 2018-02-28

**Authors:** William B. Monahan, David M. Theobald

**Affiliations:** 1 National Park Service, Inventory and Monitoring Division, Fort Collins, Colorado, United States of America; 2 Conservation Science Partners, Inc., Fort Collins, Colorado, United States of America; Universita degli Studi di Napoli Federico II, ITALY

## Abstract

We evaluate the world terrestrial network of protected areas (PAs) for its partnership potential in responding to climate change. That is, if a PA engaged in collaborative, trans-boundary management of species, by investing in conservation partnerships with neighboring areas, what climate change adaptation benefits might accrue? We consider core tenets of conservation biology related to protecting large areas with high environmental heterogeneity and low climate change velocity and ask how a series of biodiversity adaptation indicators change across spatial scales encompassing potential PA and non-PA partners. Less than 1% of current world terrestrial PAs equal or exceed the size of established and successful conservation partnerships. Partnering at this scale would increase the biodiversity adaptation indicators by factors up to two orders of magnitude, compared to a null model in which each PA is isolated. Most partnership area surrounding PAs is comprised of non-PAs (70%), indicating the importance of looking beyond the current network of PAs when promoting climate change adaptation. Given monumental challenges with PA-based species conservation in the face of climate change, partnerships provide a logical and achievable strategy for helping areas adapt. Our findings identify where strategic partnering efforts in highly vulnerable areas of the world may prove critical in safeguarding biodiversity.

## Introduction

The purpose of protected areas (PAs) is to conserve species by preserving crucial habitats, but different socio-political boundaries separating individual PAs fragment and diminish their conservation value [1–3]. Species are now also moving their distributions in response to climate change [4], and while many static PAs are critically important for realizing these movements [5,6], they are not all equally well positioned to accommodate climate-induced changes to species’ geographic ranges [7,8]. Other areas beyond the current network of PAs are needed to ensure climate change adaptation [9,10]. Dynamic or floating PAs have been advanced as a potential solution [11], but implementation would require a fundamental paradigm shift in conservation planning, which we may not have time for given the rate and magnitude of climate change. An intermediate solution between expanding vs. moving PAs is to partner or coordinate conservation actions of existing PAs with surrounding areas, thus forming what we define as “conservation partnerships”, in order to increase the effective scale of conservation [12]. However, the benefits of such partnerships for promoting climate change adaptation have not been quantified globally, which limits discussion of their conservation utility and precludes identification of strategic partnering opportunities.

Partnerships stand to increase the conservation of species by ensuring that conservation management and planning occur at biologically relevant scales, a critical need in addressing the adverse effects of socio-political boundaries on conservation [2,13]. For example, partnerships may accommodate species movements in response to climate change by ensuring that emigrants from one area are accepted and conserved as immigrants in another area. In other situations partnerships may recognize potential sources of colonization and ensure they are being conserved in ways to support future emigration. Such actions are examples of a climate change adaptation strategy that aims to extend “best practice” principles of conservation biology [10].

When responding to rapid climate change, fine-filter (e.g., individual species) and coarse-filter (e.g., biodiversity) conservation targets often converge on similar conservation actions, and many “hybrid” approaches adopt a coarse-filter prioritization of locations to selectively invest in reducing species’ vulnerabilities to climate change [14]. In a coarse-filter approach, the probability of successfully matching or aligning management scales with those of biological scales, to conserve biodiversity, increases with area and environmental heterogeneity. Broader environmental gradients manifest over larger areas counter the area-heterogeneity tradeoff [15] and increase the likelihood that more species’ area and ecological niche requirements are met. Increasing climatic variation on the landscape also decreases the distance species must move in order to keep pace with climate change [16]. Public-private conservation cooperatives favor these factors (e.g., conservation easements for non-PAs). Additional policy mechanisms include both intra- [17] and inter-governmental (e.g., Waterton-Glacier International Peace Park) conservation coordination.

Despite such opportunities, real challenges and costs exist with partnering, especially over large areas with a large number of potential partners [18]. This quest for balancing conservation gains with practical challenges prompts four overarching questions: (i) what are the established and successful scales for partnering?, (ii) how could protected areas benefit from investing in such partnerships?, (iii) who should a protected area partner with in order to gain the benefits?, and (iv) to assist global prioritization of areas threatened by climate and other forms of environmental change, where would partnerships best add to the conservation of world biomes and ecoregions that vary in their level of development risk [19] and vulnerability to climate change [20,21]?

## Materials and methods

### Protected vs. non-protected areas

Following the International Union for Conservation of Nature (IUCN), the United Nations Environmental Programme, and World Database on Protected Areas (WDPA) [22], we considered a PA to be “a clearly defined geographical space, recognised, dedicated and managed, through legal or other effective means, to achieve the long term conservation of nature with associated ecosystem services and cultural values” [23]. This definition encompasses a wide range of PAs, including “national protected areas recognised by the government, areas designated under regional and international conventions, privately protected areas and indigenous peoples’ and community conserved territories and areas” [23]. We did not select PAs based on particular IUCN Protected Area Management Categories, for “the absence of a management category does not in any way reduce the importance of a protected area, nor does it imply that the site is not being adequately managed or should be excluded from analyses” [23]. Non-PAs were defined as all terrestrial areas that were not part of the WDPA. Our non-PAs included “other effective area-based conservation measures” [23], but we did not utilize these from WDPA because “there is as yet no agreed methodology to identify these areas, and there is no global database that compiles records of all such sites” [23].

PAs from WDPA were first transformed from vector (polygon) to raster at 250 m resolution and then aggregated to 1 km resolution. The aggregation was performed in a way where the presence of only one PA cell at 250 m resolution (of 16 possible) was required to assign presence at 1 km^2^. This allowed us to retain a large number of small PAs (< 0.5 km^2^) in our global analysis. We rasterized a total of 157062 PAs using this method; 26809 PAs (17%) did not meet the 250 m resolution threshold (i.e., were less than 0.0625 km^2^ in area).

### Biodiversity adaptation indicators

We analyzed five indicators we argue are strong surrogates of biodiversity adaptation, which would be important for facilitating climate change adaptation of species, and are quantifiable using existing data over all terrestrial areas of the world. The total amount of land and freshwater area devoted to conservation is one such indicator, where larger areas increase protection for biodiversity [24]. Two additional indicators are latitudinal and elevational range, recognizing that species’ distributions are often (but not always [25]) shifting pole-ward in latitude and upward in elevation in response to climate change [4]. Because inland aquatic and riparian associated species are especially imperiled [26] and constrained to a relatively small portion of world terrestrial areas, we include a fourth indicator―relief-weighted hydrologic area, which estimates the amount of area on and immediately adjacent to the hydrologic network, weighted by the elevational relief. Finally, to evaluate the feasibility of species moving in response to climate change, climate-area velocity—similar to climate change velocity [16] but quantifiable at large landscape scales—measures the increase in area required to maintain the current climate space in the future (see below).

The five indicators were calculated using the PAs and non-PAs (above), as well as a digital elevation model [27], hydrologic network data [28], and climate data for annual mean temperature and annual precipitation [29]. Estimates of future temperature and precipitation were based on the ensemble average of 17 individual climate models available through the Coupled Model Intercomparison Project Phase 5 (CMIP5): ACCESS1-0, BCC-CSM1-1, CCSM4, CNRM-CM5, GFDL-CM3, GISS-E2-R, HadGEM2-AO, HadGEM2-CC, HadGEM2-ES, INMCM4, IPSL-CM5A-LR, MIROC-ESM-CHEM, MIROC-ESM, MIROC5, MPI-ESM-LR, MRI-CGCM3, NorESM1-M. Although an ensemble average masks variability among individual models, and individual model runs, this source of variability in climate-area velocity is generally small in relation to the spatial variability in climate across our various partnership scales (see results). CMIP5 models were downscaled and calibrated (bias corrected) using a 1950–2000 baseline. We considered a 2061–2080 future (referenced as 2070) and Representative Concentration Pathway (RCP) 8.5 W/m^2^. This RCP was selected because it is considered “business as usual” for greenhouse gas emissions and thus provides the most accurate estimate of our current climate change trajectory.

### Potential partnership areas

We measured the five biodiversity adaptation indicators across a range of spatial scales that bracket existing and successful conservation partnerships around the world, defined by the maximum linear distance (*r*) between pairs of potential partnering locations. Six radii (*r* values; 3, 9, 27, 81, 243, 729 km) were chosen to bracket a range of partnership scales (29, 254, 2290, 20611, 185502, 1669521 km^2^) encompassed by a suite of example established partnerships: e.g., Primeval Beech Forests of the Carpathians and the Ancient Beech Forests of Germany, Slovakia, and Ukraine (~290 km^2^); Tongariro National Park, New Zealand (~800 km^2^); Canadian Rocky Mountain Parks, Canada (~23,000 km^2^); Greater Yellowstone Coordinating Committee, USA (~91,000 km^2^), Yellowstone to Yukon Conservation Initiative, USA and Canada (~1,300,000 km^2^). The moving window calculations involving values of *r* were performed at a spatial resolution of 1 km^2^ on all continents and islands, except Antarctica, for both PAs and non-PAs.

We performed the moving window analysis for two partnership scenarios. Importantly, the partnerships identified in these scenarios are considered *potential* because in most instances they are not likely *realized*, but data on existing partnerships are uncommon so we cannot say conclusively which areas may actually be engaged in conservation partnerships. First, in an idealized scenario, we considered PAs and non-PAs combined (“PA & non-PA partnerships”). Ecologically this scenario is desired because it selects potential partnership areas irrespective of socio-political boundaries [2]. However, it is challenging to operationalize, so a second scenario examined potential partnerships only with respect to PAs (“PA partnerships”), recognizing that PA partnerships are favored by nature of PAs sharing a common mission to protect biodiversity and ecological processes. To address the selfish question of “what does my PA stand to gain by partnering?”, we included a null model of no partnering that took two forms, one in which no 1 km^2^ cells partnered with any other areas (“null pixel model”), plus a second where partnerships occurred only among 1 km^2^ cells of each administratively distinct PA (“null PA model”).

### Calculations

All calculations involved circular moving windows, but the partnership scale defined by the radius (*r*) could be modified for other shapes. Our values for *r* were chosen to yield window sizes that encompass six orders of magnitude (from 1 to 1669521 km^2^).

In the moving window, where partnership scale for the focal center pixel was defined by *r*, land and freshwater area (*A*) was calculated simply by tallying the number of target (PA or non-PA) pixels (*p*):

$$A=\sum_{i=1}^{n}p_{i}$$

Latitudinal range (*L*) was calculated as the maximum minus minimum latitude (*l*) of target pixels, irrespective of longitude:

L=max(l)−min(l)

Elevational range (*E*) was calculated as the maximum minus minimum mean elevation (*e*) of target pixels:

E=max(e)−min(e)

Relief-weighted hydrologic area (*H*) was used to capture the importance of valley-bottoms that provide landscape elements, such as riparian areas, across which aquatic and riparian-associated species and ecological processes can move and adapt to climate change [30]. For each target pixel located on the hydrologic network within the moving window, we subtracted its elevation by the minimum elevation in the window, and then summed the relief values:

$$H=\sum_{i=1}^{n}e_{i}-min(e)$$

**Fig 1:**
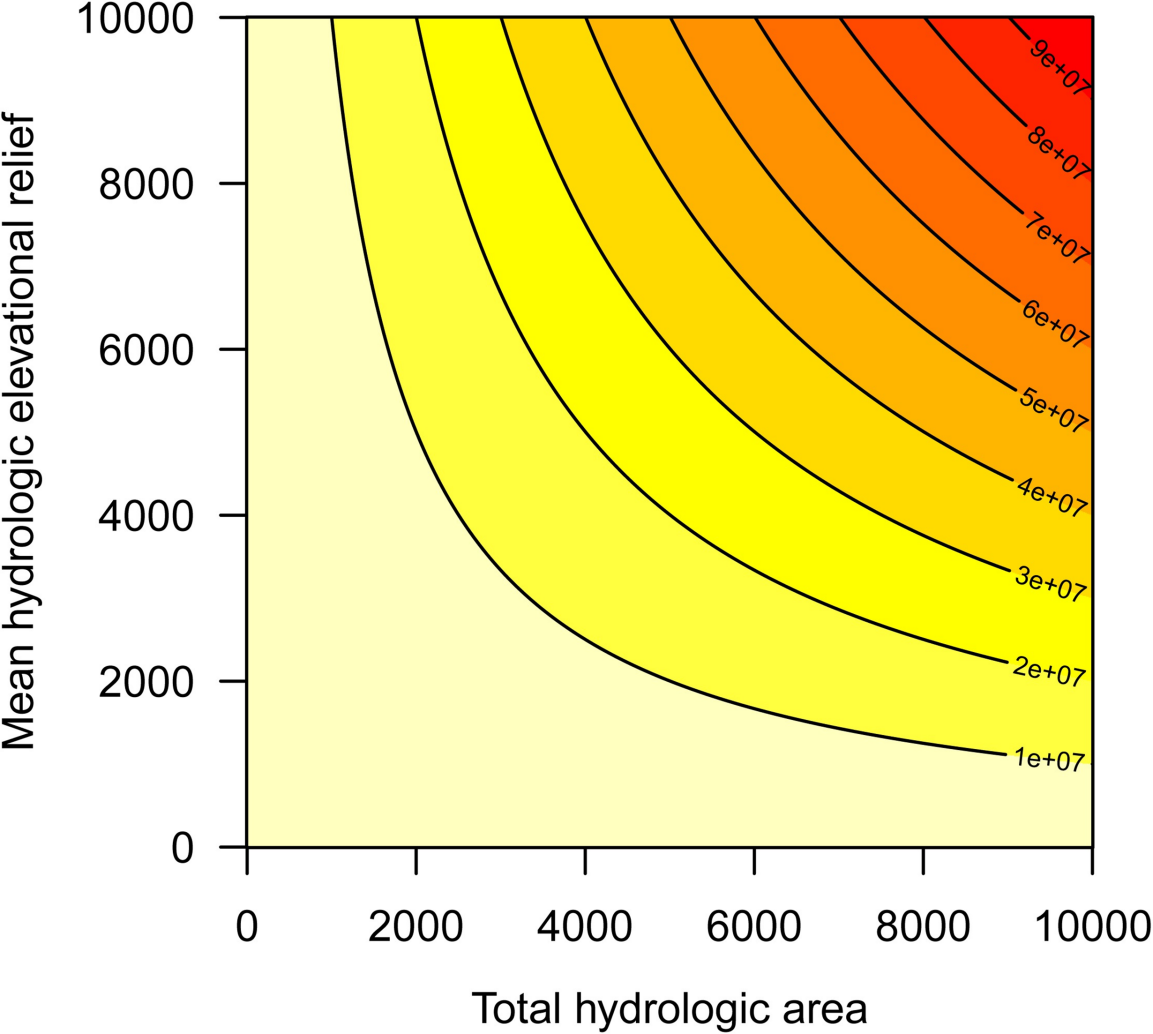
Relief-weighted hydrologic area (contours) is a function of the total hydrologic area multiplied by the average elevational relief. Protected areas tend to have large relief-weighted hydrologic area at small scales or extents because they have broad elevational ranges.

Higher values indicate greater relief and a denser hydrologic network (Fig 1). For purposes of interpreting *H*, we treated the quantity [*e*_*i*_−min(*e*)] as a unitless weight (i.e., multiplied by each target pixel on the hydrologic network). This resulted in *H* having units of area (km^2^). In addition, because at this spatial resolution we were effectively capturing terrestrial areas *near* hydrologic features, rather than the hydrologic features *per se*, we considered *H* to be more a measure of valley bottom area rather than hydrologic area. Hence, *H* includes many likely riparian areas, but we did not differentiate riparian vegetation at these locations.

Climate-area velocity was used to capture the importance of baseline climatic variation over a wide range of moving windows. We calculated climate-area velocity for both annual mean temperature and annual precipitation. While the following method and equation for calculating climate-area velocity is presented for temperature, the same procedure was used for precipitation, and it is readily extensible to other climate variables. The original climate change velocity calculation utilized the average maximum slope technique to calculate baseline climatic variation in a very small, fixed moving window (*r* = 1 pixel) [16]. However, that calculation for the denominator of climate change velocity cannot be easily employed for large moving windows (>> 3 × 3 pixels). Hence, for a given climate variable such as annual precipitation or annual mean temperature (*T*) evaluated over some time interval (*t*), we instead used its standard deviation (*σ*) across target pixels (a constant of 0.1 was used to avoid division by zero; the same constant was also used in the null model involving zero partners), and because of this change we termed the resulting measure “climate-area velocity” (*V*):

V=|ΔTΔt¯|σ(T)

**Fig 2:**
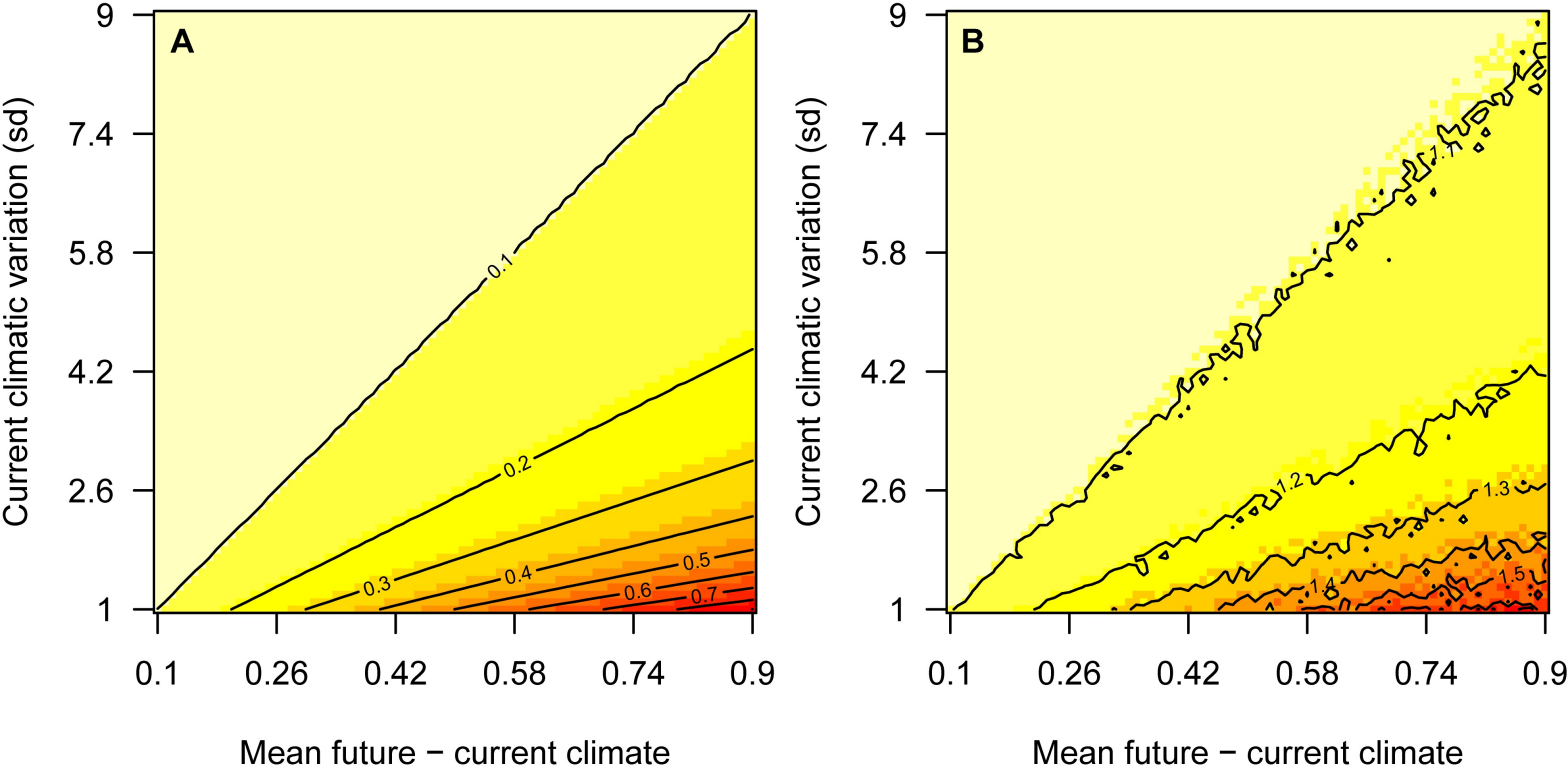
Climate-area velocity, illustrated for annual mean temperature. (A) Climate-area velocity (contours) is the factor by which area needs to increase in order to maintain the current climate space over time. (B) Simulated climate space over time. Using a sample size of 1000 and a range of plausible values for the numerator (x-axis) and denominator (y-axis) of climate-area velocity, we derived two random normal distributions (one for current climate, the other for future) and calculated the number of samples in the future that fell within the range of current values. We divided that number by the starting sample size, resulting in values (contours) where future climates contain areas that are, on average, 1.1x the size of the current climate space. Note: These contours account for the current climate space (value of 1.0), whereas in the left plot they do not; factors in the left plot may be adjusted to account for the current climate space by adding 1. We compared this simulated distribution to an empirical one derived from 1000 pixels sampled randomly from all possible protected area and non-protected area pixels (mean = 1.2x). Thus, climate-area velocity provides an estimation of the factor required to increase landscape area or context by an amount sufficient to encompass the current climate space in the future.

Where the numerator of *V* is averaged over the moving window. If *t* has units of years and *T* is °C (or mm, for annual precipitation), the units for *V* are yr^-1^ (*V* for annual precipitation has the same units), which may be interpreted as the factor by which partnership area needs to increase in order to maintain the current climate space over time (Fig 2). Furthermore, because *σ(T*) is calculated with respect to area, *A* (e.g., km^2^), the denominator of *V* could also be *σ(T*)/*A*, and *V* would have units of km^2^/yr (i.e., the area equivalent of climate change velocity, km/yr). Comparing *σ(T*)/*A* under partnerships (*r* > 0 km) vs. the null model (*r* = 0 km), the denominator could also be *Δσ(T*)*/ΔA*.

### Statistical analysis

#### What are established and successful scales for partnering?

We used established and successful conservation partnerships [18] to estimate the range of spatial scales or extents that have a proven track record of balancing conservation gains with partnership costs and complexity. Existing partnerships spanned jurisdictional, political, and watershed boundaries. We summarized the range of partnership scales using the 5^th^, 50^th^ (median), and 95^th^ percentiles of total partnership area.

#### How could protected areas benefit from partnerships?

We used the median size of established and successful partnerships (above) as our basis for evaluating how each of the two partnership scenarios compared to the two null models, as well as how the two null models compared to one another. At this scale, we considered the following five comparisons (expressed as factors): (i) PA null vs. pixel null, which provides an estimate of the climate change adaptation benefits of PAs, compared to a situation in which every pixel on the landscape acts independently; (ii) PA partnerships vs. pixel null; (iii) PA partnerships vs. PA null; (iv) PA & non-PA partnerships vs. pixel null; and (v) PA & non-PA partnerships vs. PA null. Comparisons ii-v are used as our basis for interpreting the benefits of partnering, where high benefits are indicated by large positive factors for 4 indicators (area, relief-weighted hydrologic area, latitudinal range, elevational range) and a large negative factor for climate-area velocity.

#### Who should protected areas partner with?

At the median scale for established and successful partnerships, we computed the percentage of total partnership area with PA partners, once for PAs and again for non-PAs. These percentages enabled us to estimate the degree to which PAs and non-PAs each tended to have PAs (rather than non-PAs) as potential partners. We summarized and interpreted results using two summary statistics: (i) the mean percentage partnership area with PA partners, and (ii) the percentage of area under the curve (AUC) where the percentage partnership area with PA partners was greater than 50%. Values of both summary statistics near 100% suggest PAs are most important for partnering, whereas values near 0% suggest non-PAs afford more partnering opportunities.

#### Where are areas with high adaptation potential under partnerships?

Lastly, we combined the five biodiversity adaptation indicators in order to estimate overall climate change adaptation potential for PAs partnering under the PA & non-PA scenario. These calculations were performed at the median scale for established and successful partnerships. Owing to differences in units among the indicators, we normalized each indicator based on its maximum global value, so that values ranged from 0 (little to no adaptation potential) to a maximum of 1 (highest global adaptation potential). Climate-area velocity was inverted in these calculations so that it corresponded in direction with the other four indicators. We averaged the normalized values across indicators and computed means by biome and ecoregion [31]; results were compared to the conservation risk index [19] and estimates of climate change vulnerability [20,21].

## Results and discussion

### What are established and successful scales for partnering?

The 5^th^, 50^th^ (median), and 95^th^ percentiles for established and successful partnerships are, respectively, 3.3 x 10^4^, 1.8 x 10^5^, and 1.7 x 10^6^ km^2^. If expressed in terms of circular areas, to match our moving window analysis, the radii would be 102, 242, and 729 km. These correspond closely to our a priori chosen radii of 81, 243, and 729 km.

**Fig 3:**
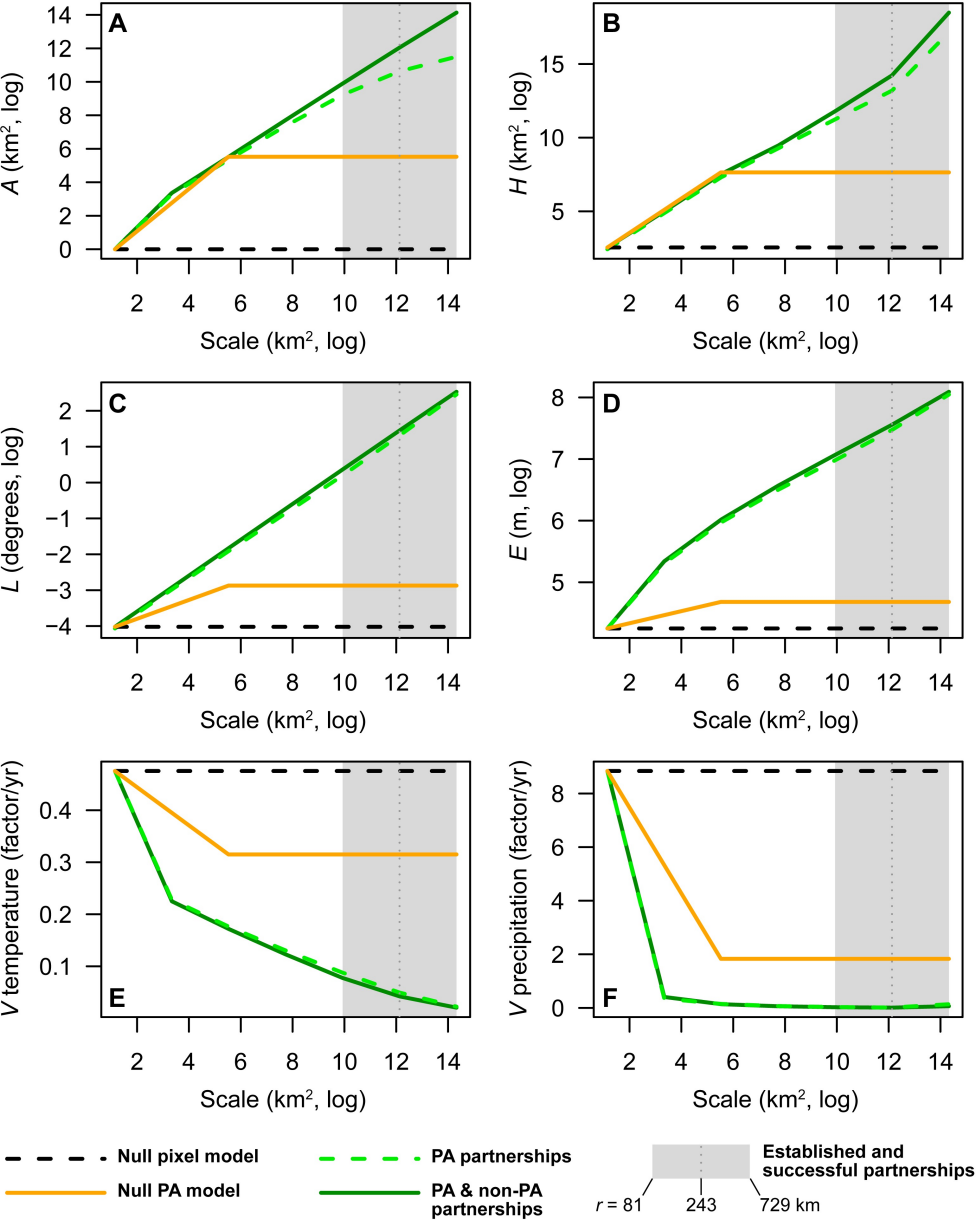
Biodiversity adaptation indicators averaged globally by partnership scale. (A) Land and freshwater area (*A*), (B) relief-weighted hydrologic area (*H*), (C) latitudinal range (*L*), (D) elevational range (*E*), and climate-area velocity (*V*) in 2070 under RCP 8.5 W/m^2^, calculated for (E) annual mean temperature and (F) annual precipitation. Following basic tenets of conservation biology, partnerships promote climate change adaptation by increasing area (A) and environmental heterogeneity (B-D). They also decrease climate-area velocity (E,F) by increasing spatial variation in temperature or precipitation. Radii (*r*) of 81, 243, and 729 km correspond closely with the 5^th^, 50^th^ (median), and 95^th^ percentiles of established and successful partnerships.

Relative to the null model where *r* = 0 km (null pixel model), established partnerships from *r* = 81 to 729 km favor climate change adaptation by increasing all indicators (Fig 3A–3D), except climate-area velocity (Fig 3E and 3F). Climate-area velocity decreases because partnerships increase spatial variability in either annual mean temperature (Table 1) or annual precipitation and thus decrease the future area needed to preserve the current climate space.

**Table 1:** Relationship between partnership scale and global averages for the numerator, °C/yr, or denominator, σ(°C), of climate-area velocity, illustrated for annual mean temperature. Climate-area velocity decreases under the two partnership scenarios because of increases in the denominator.

Partnership	PA partnerships		PA & non-PA partnerships	
scale radius (km)	°C/yr	σ(°C)	°C/yr	σ(°C)
3	0.05	0.28	0.05	0.29
9	0.05	0.47	0.05	0.49
27	0.05	0.70	0.05	0.75
81	0.05	1.04	0.05	1.12
243	0.05	1.67	0.05	1.78
729	0.05	2.80	0.05	2.98

The range of partnership scales from *r* = 81 to 729 km encompasses a number of established and successful conservation partnerships from around the world [18], suggesting it is a practical and achievable scale for conservation planning and coordination. At the same time, many successful partnerships are “old” in a climate change sense—having been established prior to climate change becoming a major issue of conservation concern—or their operational scales were set without the benefit of knowing precisely where and how climate change adaptation may favor biodiversity. In this context it is significant that our results for the biodiversity adaptation indicators at the scales associated with established and successful partnerships are considerably higher than (land and freshwater area, relief-weighted hydrologic area, latitudinal range, elevational range) and less than (climate-area velocity) those associated with the null PA model (Fig 3). These results suggest that climate change adaptation gains through partnering may be substantial. Less than 1% of world terrestrial PAs are greater than or equal to a circular area where *r* = 81 km (i.e., the lower end, or 5^th^ percentile, of established and successful partnerships), further indicating that most PAs could benefit by scaling up their conservation actions through partnerships.

### How could protected areas benefit from partnerships?

Our results summarizing the benefits to partnering, using the median scale associated with established and successful partnerships (*r* = 243 km), are shown in Table 2. Compared to the null pixel model, the biodiversity adaptation indicators under the null PA model are 1.5x to 251x greater in magnitude. This suggests that PAs pursuing climate change adaptation, even in isolation as distinct administrative units, often reap adaptation benefits because of their size. However, these benefits are small in relation to those gained under partnerships. PA partnerships are 6.4x to 257x greater in magnitude than the null PA model. Thus, climate change adaptation should not simply require the addition of more land irrespective of who owns and manages the area. PA partnerships that seek to coordinate conservation practices among different PAs (e.g., because the PAs share conservation targets) stand to dramatically increase the effective scale of conservation. Furthermore, these benefits are maintained or even increased if PAs partner not only with other PAs but also non-PAs; under the PA & non-PA partnerships scenario the biodiversity adaptation indicators are 7.5x to 724x magnitude greater than the null PA model.

**Table 2:** Differences between each of two partnership scenarios and two null models, at the median scale or extent associated with established and successful partnerships (*r* = 243 km). Numbers report the factor increase or decrease between each scenario in a pair.

Scenarios	Area	Relief-weighted hydrologic area	Latitudinal range	Elevational range	Climate-area velocity (temperature)	Climate-area velocity (precipitation)
Null PA model vs. Null pixel model	251	164	3.2	1.5	-1.5	-4.8
PA partnerships vs. Null pixel model	41241	42073	207	25	-9.6	-571
PA partnerships vs. Null PA model	164	257	66	16	-6.4	-118
PA & Non-PA partnerships vs. Null pixel model	170612	118553	238	27	-11.3	-773
PA & Non-PA partnerships vs. Null PA model	680	724	76	18	-7.5	-160

Contrasting results for the two partnership scenarios (Table 2), each relative to the two null models, the two area indicators (land and freshwater area, relief-weighted hydrologic area) both exhibit especially large differences under PA & non-PA partnerships. Meanwhile, results are more similar for both elevational range and climate-area velocity. These patterns are due to a combination of two factors. First, although PAs occupy less area than non-PAs, PAs also tend to occur in higher mountainous elevations compared to non-PAs [32], which increases elevational range as well as the denominator of climate-area velocity. Second, because PAs only occupy about 14% of terrestrial areas globally [24], in a given moving window PAs will only include a fraction of all pixels. This results in lower estimates of area, compared to those obtained using all pixels (PA & non-PA partnerships). Relief-weighted hydrologic area is noteworthy because the discrepancy between the two partnership scenarios is so large (Table 2). Although PAs can promote climate change adaptation by maximizing hydrologic elevational relief at smaller partnership scales, they can increase it tremendously more by partnering with non-PAs on a much larger hydrologic network. In other words, compared to terrestrial biodiversity, aquatic and riparian-associated biodiversity likely require partnering at broader scales.

### Who should protected areas partner with?

**Fig 4:**
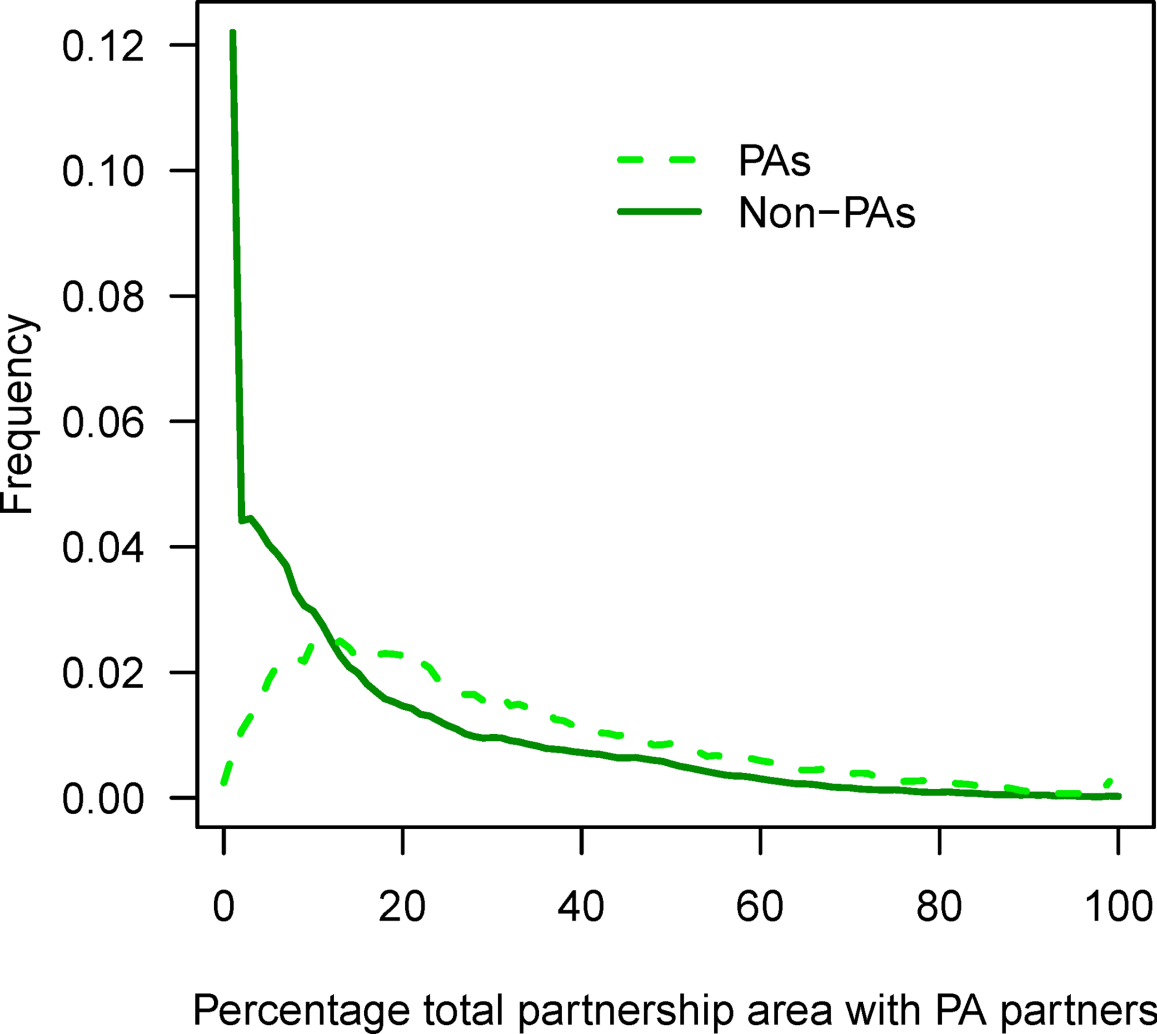
Percentage of total partnership area with PA partners, measured at the median scale or extent associated with established and successful partnerships (*r* = 243 km).

PAs comprise a relatively small amount of partnership area (Fig 4). In terms of PA partnerships, partnership area with other PAs is, on average, 30%, whereas for non-PAs it is 19%. Furthermore, the amount of partnership area with ≥ 50% PA partners is 18% for PAs and 8% for non-PAs. Hence, non-PAs—areas that do not currently have as their primary purpose to support the long-term conservation of nature—are a significant potential contributor to partnerships worldwide. These results highlight the importance of thinking beyond the current world network of PAs when planning for climate change adaptation. Some non-PAs are publically owned and managed, so policies that facilitate governmental coordination offer opportunities to scale up conservation of PAs with other public non-PAs. However, many non-PAs are private (e.g., private lands comprise approximately 60% of the US), so additional policies are needed to encourage public-private conservation cooperation. Nonetheless, with approximately 86% of world terrestrial areas in non-PA status, the principal challenge for promoting climate change adaptation via partnerships lies in formulating incentive-based mechanisms for developing and sustaining partnerships among non-PAs.

### Where are areas with high adaptation potential under partnerships?

**Fig 5:**
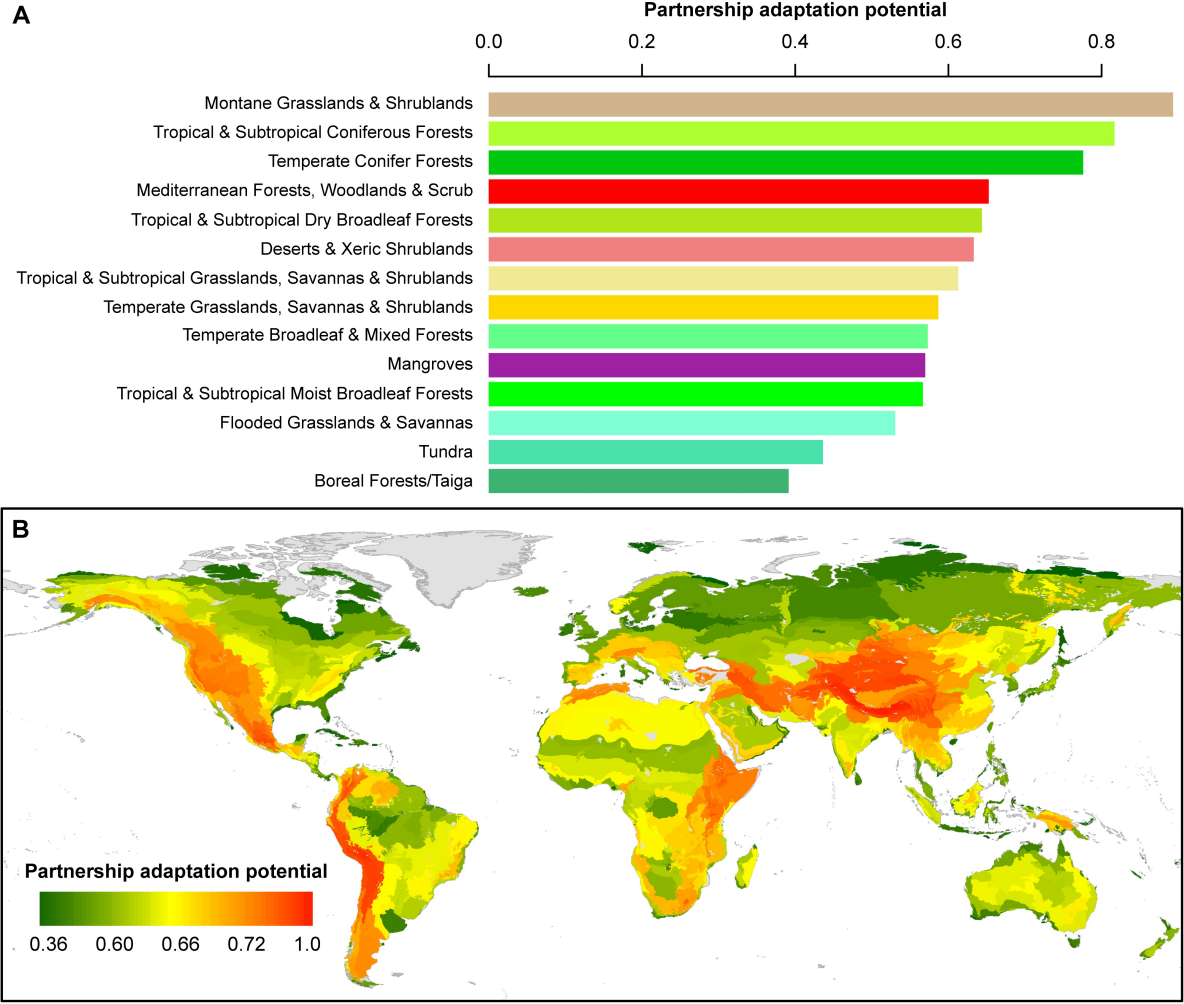
Climate change adaptation potential under conservation partnerships. (A) Biomes, (B) ecoregions.

Partnerships offer opportunities to promote the climate change adaptation of biodiversity in areas that vary by level of development risk (conservation risk index, CRI) [19] and climate change vulnerability [20,21]. Globally the most threatened biome from development and lack of protection is temperate grasslands, savannas & shrublands [19], but it also exhibits a moderate level of adaptation potential under partnerships (0.59, Fig 5A). As the world’s lowest ranked biome for percentage PA (4.4%), partnerships with non-PAs are especially important for promoting the biodiversity adaptation indicators on remaining natural lands, which have yet to be significantly transformed for human uses. Conversely, two of the three highly ranked biomes under partnerships (montane grasslands & shrublands; and temperate conifer forests; Fig 5A) possess low CRI, while the third (tropical & subtropical coniferous forests) has a moderate CRI [19]. One of these (montane grasslands & shrublands) has the highest percentage PA in the world (26.5%) and thus stands to benefit especially from PA partnerships. Similarly, ecoregions with high adaptation potential under partnerships and high vegetation intactness, but low climate stability [20], include northwestern North America (Canada and Alaska, USA) and northern Tibetan Plateau (Fig 5B). Other areas with high partnership adaptation potential and a large number of species vulnerable to climate change (>50 species) [21] include the Sierra Nevada Mountains (California, USA), south-central Mexico, the Andes and western ecoregions in South America, north-central Africa, northern Arabian Peninsula, and the Himalayas and Tibetan Plateau steppe (Fig 5B).

### Future directions

Our work contributes to the IPCC framework for implementing climate change adaptation by helping identify management options [33]. PA partnerships with other PAs and non-PAs facilitate adaptation by promoting basic tenets of conservation biology. Our results emphasize the potential for existing PAs to steward a future for biodiversity in the face of climate change by serving as anchors for broader conservation partnerships. Such partnerships stand to play a pivotal role in the conservation of individual species and biodiversity at local to regional scales. Additional work is required to improve our biodiversity indicators and monitor how they change, especially taking into account invasive species and human land uses and how they impact our ability to restore habitats and conserve biodiversity [34,35]. Related, because not all species are equally poised to track changes in climate on the landscape [36] (e.g., due to variation in dispersal, demographics, life history traits, etc.), monitoring species’ responses would provide direct insights into the effectiveness of conservation partnerships, yielding results that further help managers strategize more proactive conservation tactics (e.g., assisted migration) or detect and prevent unintentional outcomes (e.g., transport of non-native species). Studies that explore the impacts of different climate change scenarios (e.g., warmer and wetter vs. drier futures) would further assist partners with conservation planning in the face of future climate uncertainty. New studies are also warranted for examining (i) partnership equality (e.g., ecological or logistical tradeoffs among partnering areas [37]); (ii) the directionality of partnerships needed under climate change (i.e., given directional changes in climate, partnerships may not be balanced and reciprocal, thus complicating incentives for partnering); (iii) the proximity, connectivity and representativeness [38] of partnering areas; and (iv) partnerships for special groups of species (e.g., marine or migratory species; species that are less able to track changes in climate). Finally, a clearer understanding is needed of the laws and policies supporting climate change adaptation in different countries [39], including the mechanisms and incentives that promote cooperation.
